# No significant effect of frequent online sexual behaviour on Pavlovian-to-instrumental transfer (PIT): Implications for compulsive sexual behaviour disorder

**DOI:** 10.1371/journal.pone.0274913

**Published:** 2022-09-30

**Authors:** Timothy J. Wells, Lucie Krejčová, Jakub Binter, James G. Pfaus, Rachel R. Horsley

**Affiliations:** 1 Center for Sexual Health and Intervention, Czech National Institute of Mental Health, Klecany, Czech Republic; 2 Department of Philosophy and History of Science, Charles University, Prague, Czech Republic; 3 Department of Psychology and Life Sciences, Charles University, Prague, Czech Republic; 4 Psychology Department, Nottingham Trent University, Nottingham, United Kingdom; Murdoch University, AUSTRALIA

## Abstract

Reward based learning is broadly acknowledged to underpin the development and maintenance of addictive behaviour although the mechanism in sexual compulsivity is less understood. Using a Pavlovian-to-Instrumental Transfer (PIT) task we tested whether the motivational aspect of conditioned Pavlovian conditioned stimulus invigorated instrumental responding in relation to specific compatible monetary rewards. Performance on the task was analysed between two groups of males based on Low (N = 38) and High (N = 41) self-report online sexual behaviour (OSB). Psychometric tests including sexual compulsivity scale and behavioural activation/behavioural inhibition (BIS/BAS) were also administered to determine the relationship between OSB and general reward sensitivity. We show clear evidence of acquisition in the Pavlovian and instrumental conditioning phases. Specific transfer effect was greater in the High-OSB group although the difference compared to the Low-OSB group was non-significant. OSB negatively correlated with both BIS and BAS indicative of introversion and low reward sensitivity. OSB positively correlated with sexual compulsivity although it is unclear whether individuals in the High-OSB group considered their behaviour either excessive or problematic. These findings contribute to the ongoing debate regarding the nature of problematic OSB. Fundamental differences in motivational characteristics and mechanism contributing to compulsive behaviour in relation to high-OSB might indicate incompatibility with behavioural addiction models. PIT was not enhanced in high-OSB by appetitive conditioning, although problematic OSB could stem from failure to inhibit actions. Further research should investigate whether aversive conditioning differentially affects responding in high-OSB individuals, potentially explaining perseverant behaviour despite negative consequences.

## Introduction

There is currently a vociferous debate about the nature of compulsive sexual behaviour as it relates to “problematic” internet pornography use. Despite positive effects arguably associated with online sexual behaviour (OSB) and the viewing of visual sexual stimuli, for example, reports of improved sex life owing to improved sexual communication and increase in sexual repertoire [1–3], excessive use can become problematic and detrimentally affect an individual’s personal life and relationships [4–6]. Excessive pornography use is currently classified under the definition of compulsive sexual behaviour disorder (CSBD) in the International Classification of Diseases (ICD-11) [7]. The reinforcing nature of sexual stimuli is often cited as a motivational factor driving such behaviour (see [8]). However, the mechanism underlying perseverant problematic OSB is equivocal. The primary aim of the present study was to test whether men who scored highly for OSB (e.g., risky cybersex and risky internet pornography use) translate reward predicting cues into goal-directed actions more readily than men who do not score highly for risky OSB.

Evidence from neurobiological studies suggest augmented responses to visual sexual stimuli in attentional and reward-related brain structures (e.g., nucleus accumbens, NAc; [9–12]. This is in line with similar activations observed for substance abuse [13, 14], gambling [15], and food related disorders [9,16], and suggests that some individuals are more sensitive to reward-related cues as drivers of behaviour. These individuals may therefore form obsessive and compulsive behaviours around those cues more readily than others. However, alternative explanations suggest that compulsive masturbation develops with pornography as means to stimulate sexual arousal [17]. If individuals struggle with this, or have guilt associated with engaging in such behaviour [18], then the development of augmented sensitization may be due to reward prediction errors or reward uncertainty [19, 20]. This would be especially potent if viewing internet pornography and masturbation was engaged intermittently (e.g., on a partial reinforcement schedule) and/or if individuals experienced unusually prolonged refractory periods following excessive masturbation resulting in several ejaculations (a state that some self-identified internet pornography “addicts” call Porn-Induced Erectile Dysfunction, or PIED) [21].

Men that have a greater frequency of pornography consumption per week are actually better able to achieve erection to visual sexual stimuli in laboratory situations relative to men with a lower frequency of pornography consumption [22]. However, men with frequent but problematic pornography consumption who also have high sexual desire show a blunted processing of visual sexual cues [23], suggesting some attempt at active inhibition. It is also possible that individuals that consume more pornography generally have attentional and reward-related brain systems that are driven naturally toward highly valued incentives. Models of conditioned behaviour posit that some individuals (goal-trackers) simply focus on reward outcome and engage in conditions that predict delivery whilst others (sign-trackers) are drawn to the cues associated with a reward to the extent that conditioned stimuli become a source of motivation themselves (see [24]). Sign-trackers are believed to be more predisposed to developing compulsive behaviour and at risk of relapse owing to the motivational incentive properties attributable to predictive cues. Although research has predominantly focused on substance misuse and pathological gambling (e.g., [25, 26]), it may follow that problematic pornography users would similarly display enhanced sign-tracking of high reward value goals.

Pavlovian reward predictive cues that become imbued with motivational salience promote the likelihood of repeated exposure [27]. In addition, positively reinforced instrumental responding increases the likelihood of repeating an action. Pavlovian Instrumental Transfer (PIT) allows these two paradigmatic elements to interact when the motivational aspect of a Pavlovian conditioned stimulus is transferred, resulting in an increase in the learned Instrumental behaviour in the presence of the cue (for a recent review see [28]. There are two subtypes of PIT that have dissociable neural substrates [29]; specific PIT occurs when rewards associated with a conditioned stimulus enhance instrumental responding for the same reward, thus elucidating the motivational drive to obtain a predicted outcome. General PIT refers to the enhancement of instrumental responding when the rewards in the two learning phases differ and is thus a measure of general arousal. PIT is a naturally adaptive process through which individuals learn to forage for resources, for example, to sate natural impulses such as hunger and thirst.

Bray et al. [30] found increased specific PIT responding in a group of typical individuals using soft drinks and concluded that transfer effects relating to reward predicting cues were not the sole preserve of problematic behaviour. Lehner et al. [31] showed that food, money, and social rewards facilitate increased instrumental responding; postulating that the strength of PIT is modulated by subjective value rather than type of associative reward. Nevertheless, PIT has proven effective describing the maladaptive process that can facilitate the development and maintenance of substance addiction [32]. Drug related cues induce craving and invigorate instrumental responding [33–36]; an effect that is more pronounced in dependent individuals [33, 35].

The propensity for sexual imagery to bias behaviour has been leveraged for some time in advertising. Male and female models are often employed to associate products with an unconditioned arousal response. Sexually explicit material accounts for an enormous proportion of internet traffic [5, 37]. With the advent of targeted advertisements and pop ups, individuals are regularly exposed to unsolicited sexual content; moreover, interacting with such content can result in algorithmic changes that provide the user with further content. However, not all individuals succumb to temptation [38] and only a small proportion may develop a compulsive habit of viewing sexual stimuli as a means of generating sexual arousal for masturbation [4, 39].

Individual differences in reward sensitivity may occur by the interaction of two motivational systems [40], behavioural activation and behavioural inhibition. These dual control systems for any motivated behaviour provide it with a beginning, middle, and end, and for sexual behaviour that can be seen in the models of Moll [41], Masters and Johnson [42], Bancroft and Janssen [43], and Toates [44]. The behavioural activation system (BAS) regulates appetitive rewards and the motives to instigate approach behaviour. The neuroanatomy associated with BAS incorporates the mesolimbic pathway, including ventral tegmental area (VTA), ventral striatum, and pre-frontal cortex regions of the reward network [45, 46]. BAS comprises three discrete dimensions: drive (reward-focused in pursuit of goals) fun-seeking (engages in spontaneous, novel, and potentially rewarding sensations), and reward-responsiveness (anticipates and responds positively to rewarding outcomes). The behavioural inhibition system (BIS) influences the end of responding along with a shifting of attention to other rewards, non-rewards, or aversive cues, and accordingly abrogates behaviour. Neuroanatomy associated with BIS is believed to include regions such as the amygdala and hippocampus [47, 48]. Individual differences in dimensions of BAS sensitivity, in particular, have been associated with addictive behaviour [49, 50]. Moreover, general reward responsiveness has been found to correlate positively with responses to sexually explicit visual stimuli [51]. As such, a positive correlation between BAS dimensions and OSB scores was expected.

The present study aimed to elucidate if a general mechanism of reward sensitivity might exist to reinforce OSB. Accordingly, we investigated the relationship between OSB and behavioural activation/inhibition. Furthermore, differences in performance on a PIT task were determined based on self-report measure of OSB use that allowed us to divide participants into low and high use groups. We used an analogue task with monetary rewards in order to determine potential differences in a general mechanism of learning that was not biased by preferences for sexually explicit stimuli. Regardless, selecting standardised stimuli for experiments involving pornography can be problematic given the variety in preferences for content and degree of explicitness [see 52]. Indeed, the effects of sexual conditioning also tend to be weak in adults [53] possibly owing to already formed preferences and their heterogeneity [54]. However, individuals that exhibit heightened reactivity to drug related cues also showed greater reward responsiveness to non-drug related reinforcement [55, 56]. As such, a difference in general reward mechanism was expected. Specifically, we anticipated that individuals with higher OSB scores would transfer more strongly than those with lower OSB scores.

## Method

### Ethics statement

Ethical approval for the study was granted by the National Institute of Mental Health Research Ethics Committee (No: 53/16). All individuals provided informed written consent to participate.

### Design

In the PIT task, prior to the transfer phase, baseline Pavlovian conditioning was established in phase 1, measured as post-conditioning increases in pleasantness ratings (-5 to +5) for conditioned Pavlovian stimuli, and baseline instrumental conditioning was established in phase 2, measured as the percentage (%) of trials where rewarding outcomes were selected over the neutral outcome. Each used a within-groups experimental design to establish post-conditioning learning. A two-sample between-groups experimental design was used to compare the magnitude of the transfer effect, measured as percentage (%) responses in the transfer phase (phase 3) of the PIT task, in low- and high- (problematic) online sexual behaviour (OSB) groups. In addition, three behavioural control groups were included in phase 3 of the PIT; Pavlovian reward, Pavlovian neutral and neutral choice controls, which were likewise measured as percentage (%) responses.

Low- and high-OSB groups were determined psychometrically, using the Internet Sex Screening Test online sexual behaviours subscale (ISST-OSB) [57], and exploratory between-groups comparisons were made of scores on the Sexual Compulsivity Scale (SCS) [58] and the Behavioural Inhibition System and Behavioural Activation System (BIS/BAS) [40] scales (see *Table 1*). In addition, the scores from the ISST-OSB, along with the aforementioned psychometric measures were used to perform exploratory correlational analyses.

**Table 1:** Participant characteristics and descriptive statistics for psychometric measures in low- and high-OSB experimental group.

	Low-OSB		High-OSB	
	*M*	*SD*	*M*	*SD*
Age	25.4	*4*.*9*	25.58	*4*.*7*
DAST-10	1.63	*1*.*35*	1.82	*1*.*43*
sMAST	.85	.*63*	1.0	*1*.*04*
ISST-OSB	**5.88**	*1*.*78*	11.82*	*2*.*8*
SCS	**14.98**	*4*.*04*	18.82*	*5*.*54*
BIS	**16.9**	*1*.*95*	15.82*	*2*.*08*
BAS Drive	8.85	*1*.*71*	8.23	*1*.*32*
BAS Fun	**9.61**	*1*.*96*	8.53*	*1*.*9*
BAS Reward	**12.39**	*2*.*06*	10.97*	*1*.*81*

Low-OSB (lower risk for problematic online sexual behaviours); High-OSB (higher risk for problematic online sexual behaviours); DAST-10 (Drug Abuse Screening Test, 10-item form); sMAST (Michigan Alcohol Screening Test, short form); ISST-OSB (Internet Sex Screening Test-Online Sexual Behaviours subscale); SCS (Sexual Compulsivity Scale); BIS (Behavioural Inhibition System); BAS (Behavioural Activation System). Asterisks indicate significant differences between low- and high-OSB groups at *p* < 0.05.

### Participants

Czech participants (*N* = 89) were recruited through Czech language advertisements placed on social media pages associated with the Sexology and Psychopathology Research Group at the National Institute of Mental Health (NIMH, Klecany, Czech Republic) and the Human Ethology Department at Charles University in Prague (Czech Republic). Prior to the study, participants were screened for drug and alcohol misuse with the Drug Abuse Screening Test (DAST-10) [59] and short version of the Michigan Alcohol Screen Test (sMAST) [60] since co-morbid substance use can confound conditioning effects [61]. Data from eight individuals were excluded for exceeding drug and alcohol cut-off scores. Data from two further participants were excluded owing to incomplete questionnaires. Remaining participants comprised 79 males that were separated into low (*N* = 41) and high (*N* = 38) OSB–see Table 1.

### Psychometric measures

A professional academic and scientific translation agency was employed to undertake the translation of psychometric scales into Czech language. This process also included backwards translation of the Czech version into English to ensure scale items had not lost or altered meanings during this process. At each stage, academics from the CSHI at the Czech NIMH with the first language of Czech or English discussed and approved the final translations as accurate versions of the English language originals.

Drug Abuse Screening Test (DAST-10): The DAST-10 [59] was used to measure problematic drug use/dependency. This unitary 10-item scale measures yes/no responses; scores of four or more indicate a ‘moderate’ level problematic drug use/dependence. The DAST demonstrates good reliability and validity [62].

Michigan Alcohol Screen Test, short form (sMAST): The sMAST [60] was used to measure problematic alcohol use/dependency. A 13-item unitary scale is used to measure yes/no responses (items 1, 4 and 5 were reverse scored) which vary from zero to 13; total scores of three or more indicate the presence of borderline alcohol use/dependence. The sMAST demonstrates good reliability and validity [60].

Internet Sex Screening Test—Online Sexual Behaviours (ISST-OSB): ISST-OSB [57] is a 34-item true/false scale comprising two subscales. The first, ISST-OSB, which measures variation in online sexual behaviours (OSB), primarily risky cybersex and use of online sexual content (25 items, total scores vary from zero to 25, with scores >9 indicative of problems such as excessive use of/spending on pornography or online sexual services, often in isolation, with negative consequences for relationships, finances, employment etc. [57]. The cut-off of 9 was used to determine experimental groups (low- versus high OSB) for the analysis of the PIT data (Table 1). ISST-OSB captures dimensions including time spent in isolation consuming sexual materials online, degree of interest in sexual materials and activities online, money spent in the pursuit of such materials and activities, as well as participation or online consumption with other parties. The ISST has been widely used with clinical samples, however limited information about its reliability and validity has been published. The second subscale, the Abbreviated Sexual Addiction Screening Test (ASAST: nine items, scores vary from zero to nine) measures general sexual addiction and compulsivity [57]. The scale was administered in full, however only responses to the 25-item ISST subscale were included in analyses, since sexual compulsivity was measured using the more widely used measure, the SCS [58].

Sexual Compulsivity Scale (SCS): SCS [58] includes a series of 10 statements concerning sexual thoughts and feelings of control. Responses (level of agreement from 1—not at all like me to 4—very much like me) are summed providing possible overall scores between 10 and 40. The SCS was administered to facilitate differentiation between effects of compulsivity and conditioning on choice behaviour in the PIT task.

Behavioural Inhibition System and Behavioural Activation System (BIS/BAS): BIS/BAS [40] measures competing approach/avoidance tendencies resulting from underlying motivational systems that are geared to activate or inhibit behaviours to obtain appetitive outcomes and avoid aversive consequences or harm. The scale includes 24 statements, with responses indicated agreement from 1 –very true for me, to 4 –very false for me. Scores are calculated separately for the BIS subscale and three BAS dimensions; BAS Drive, BAS Fun-seeking and BAS Reward-responsiveness, which concern the motivation to pursue goals, seek novel or spontaneous sensations, and savour positive experiences, respectively.

### Behaviour: Pavlovian-to-instrumental transfer task (PIT)

**Fig 1:**
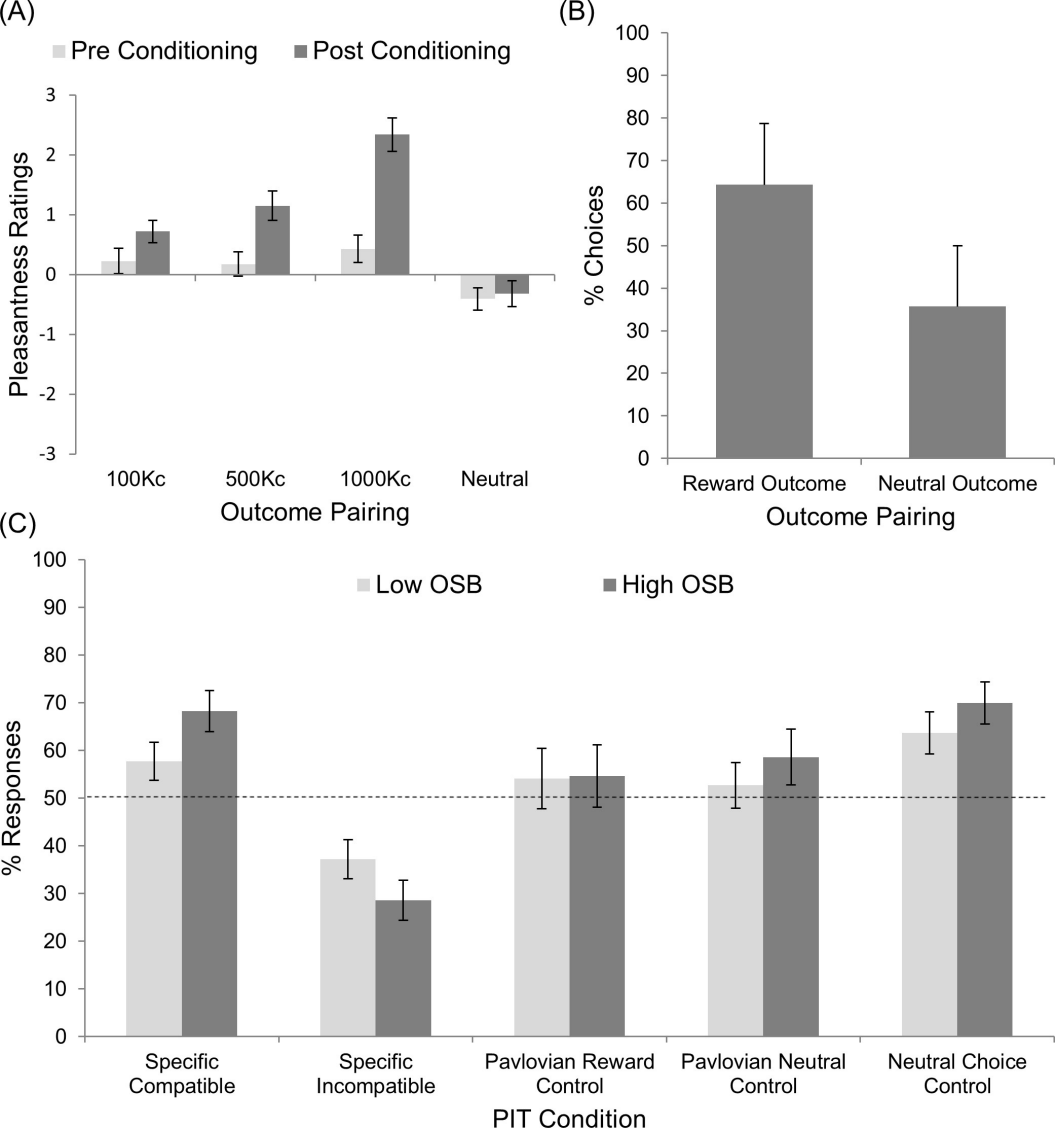
The Pavlovian-to-instrumental transfer (PIT) task. Exemplar stimulus slides for each of the three experimental phases are shown (A) phase 1, Pavlovian conditioning; (B) phase 2, instrumental conditioning; and (C) phase 3, Pavlovian-to-instrumental transfer. Within each experimental phase, time (ms) is represented from top-to-bottom, with timings for stimulus slide presentation shown on the left. The struck banknote in the transfer phase denotes that no monetary or neutral outcome was delivered in this phase.

The behavioural task was adapted from a PIT variant used in humans by Bray et al. [30]. The task was administered using *E-prime* 2.0 (Psychology Software Tools, Pittsburgh, PA) experimental presentation software. Participants were initially instructed to pay attention to the relationship between cue-outcome and action-outcome associations throughout the entire experiment. The PIT task variant that was implemented comprised three phases: phase 1 –Pavlovian conditioning; phase 2 –instrumental conditioning; and phase 3 –Pavlovian-to-instrumental transfer (the transfer phase). Fig 1 shows a visual representation of the three phases and Table 2 provides specifics on the reinforcement schedule and outcomes.

**Table 2:** Trial composition during conditioning and transfer phases of the Pavlovian-to instrumental transfer task.

Phase		Transfer Condition	No of Trials	Stimulus (Outcome)		
1. Pavlovian Conditioning			6	*S1 (100 Kc)*		
			6	*S2 (500 Kc)*		
			6	*S3 (1000 Kc)*		
			6	*S4 (Neutral)*		
2. Instrumental Conditioning			6	*R1 (100 Kc)*	*or*	*R2 (500 Kc)*
			6	*R1 (100 Kc)*	*or*	*R3 (Neutral)*
			6	*R1 (100 Kc)*	*or*	*R4 (Neutral)*
			6	*R2 (500 Kc)*	*or*	*R3 (Neutral)*
			6	*R2 (500 Kc)*	*or*	*R4 (Neutral)*
			6	*R3 (Neutral)*	*or*	*R4 (Neutral)*
3. Pavlovian to Instrumental Transfer		**OSC**/OSI	12	***S1 + R1 (—)***	*or*	*S1 + R2 (—)*
		OSI/**OSC**	12	*S2 + R1 (—)*	*or*	***S2 + R2 (—)***
		PRC	12	*S3 + R1 (—)*	*or*	*S3 + R2 (—)*
		PNC	12	*S4 + R1 (—)*	*or*	*S4 + R2 (—)*
		NCC	12	*S4 + R3 (—)*	*or*	*S4 + R4 (—)*

Phase 1- Pavlovian conditioning: Four unique Pavlovian stimuli were created using varying configurations of a red rectangle, triangle and circle. To control for baseline preferences for the Pavlovian stimuli, these configurations were designed to contain identically sized and coloured components, and to be meaningless; however, in pilot testing, we found that participants found it difficult to learn the associations. The same identical shapes were reconfigured so that their aggregates could be more easily verbalised by participants as something meaningful e.g., ‘house with sun’, which improved performance in this phase.

During Pavlovian conditioning, stimuli were presented in the centre of the screen for 1750 *ms* before an outcome screen for 3000 *ms* (Fig 1A). Each stimulus was paired with a monetary reward of different denominations (100, 200, and 1000 Kc banknotes) of currency or with a well-known brand of toy money at its lowest denomination as the neutral outcome (Table 2). Pavlovian stimuli were followed by monetary reward at a 3:2 (reward/no-reward) ratio. Each trial was separated with a blank ‘wait’ screen (which varied randomly between 1000 and 3000 *ms*). Each stimulus was presented 6 times in a randomised order, yielding a total of 24 trials.

Phase 1 (24 trials) was Pavlovian conditioning, phase 2 (36 trials) was instrumental conditioning, and phase 3 (60 trials) was the transfer test phase. There were four Pavlovian stimuli-outcome pairings and there were four possible instrumental (button options) response-outcome options that were presented in successive trials as a dichotomous choice. Outcomes during phases 1 and 2 were images of 100, 200 or 1000 kc banknotes, or a toy banknote as the neutral outcome. No outcome was presented in extinction during the transfer phase 3. S1-S4 (Pavlovian stimuli); R1-R4 (response button options); OSC: outcome specific compatible; OSI: outcome specific incompatible; PRC: Pavlovian reward control; PNC: Pavlovian neutral control; NCC: neutral choice control

To assess Pavlovian conditioning, cue evaluation involved participants rating the pleasantness of Pavlovian cues both pre- and post-conditioning phase. Visual cues were presented on screen for 1750 ms followed by a visual scale from -5 (unpleasant) to 5+ (pleasant). The ARROW keys could be used to move up and down the scale whilst pressing ENTER to confirm a highlighted number selected. Stimuli were presented in a randomised order.

Phase 2- Instrumental conditioning: During Instrumental conditioning (Fig 1B), participants were required to choose between two button options. A row of four blank squares appeared in the lower third of the screen of the screen which corresponded to four adjacent keys on a keyboard, in the same spatial arrangement. Each of the four responses was associated with a specific outcome (Table 2). Unlike during Pavlovian conditioning, here only two responses were associated with monetary values whilst two related to the same neutral outcome (toy money). In each trial, two of the blank squares were replaced with coloured images denoting the two responses available for selection. Instrumental cues were presented on screen for 1750 *ms* before an outcome screen for 3000 *ms*. Response-outcome pairings were partially reinforced on a 2:3 ratio. Each trial was separated with a blank ‘wait’ screen (which varied randomly between 1000 and 3000 *ms*). There were six configurations of response-outcome pairings, which were each presented six times in a randomised order yielding a total 36 trials.

Phase 3-Pavlovian-to-instrumental transfer: Bray et al. [30] conducted an additional training phase that comprised mixed Pavlovian and instrumental trials in order to consolidate learning prior to the transfer phase. A pilot showed that mixing trials created confusion and resulted in poor transfer performance. As a result, here instrumental conditioning was followed immediately by the transfer phase. Participants were instructed to provide a response during the simultaneous presentation of Pavlovian and instrumental cues. Unlike earlier conditioning phases, no monetary or neutral outcomes occurred during transfer trials constituting extinction (Table 2). Cues were presented on screen for 1750 *ms* before a blank outcome screen for 3000 *ms* (Fig 1C). Each trial was separated with a blank ‘wait’ screen (which varied randomly between 1000 and 3000 *ms*). There were five conditions in the transfer phase that were each presented 12 times in a randomised order over a total of 60 trials.

Outcome-specific transfer occurred when Pavlovian cues biased participants towards the instrumental response corresponding with the common conditioned outcome(s). With specific transfer, Pavlovian cue S1 was only associated with the same outcome as instrumental response R1; and Pavlovian cue S2 was only associated with the same outcome as instrumental response R2. With specific incompatible transfer, participants could also choose the incompatible reward response, for example, by selecting response R1 despite previously established associations with Pavlovian cue S2 or by selecting response R2 despite previously established associations with Pavlovian cue S1.

In the Pavlovian reward control condition, participants chose between the two rewarding instrumental response options (R1 and R2) whilst the Pavlovian cue S3 was associated with an outcome seen during Pavlovian conditioning, but that was absent during the instrumental conditioning phase; thus, neither response option was compatible. In the Pavlovian neutral control, participants chose between the two rewarding instrumental response options (R1 and R2) whilst the Pavlovian cue S4 was associated with a neutral outcome (toy money). Again, neither instrumental response was compatible and participants might be expected to bias response selection toward instrumentally conditioned rewards. Finally, in the neutral choice control, Pavlovian cue (S4) was associated with the same neutral outcome as both instrumental response options (R3 and R4).

### Data analyses

All analyses were conducted in PASW(SPSS)® ver. 22 (IBM). All statistical tests had the alpha criterion of .05, two-tailed (unless otherwise stated).

A mixed 4 x 2 x 2 ANOVA was used to evaluate the effectiveness of Pavlovian conditioning in phase 1; incorporating repeated measures in pleasantness ratings for each of the four cues pre- and post-conditioning and a test of difference between OSB groups. If the Pavlovian cues had been associated successfully with ‘rewarding’ monetary outcomes, then pleasantness ratings should be higher post-conditioning, and we expect a stepped pattern in line with increasing monetary value.

A mixed 2 x 2 ANOVA was used to evaluate the effectiveness of instrumental conditioning in phase 2; incorporating repeated measures (the pooled number of choices a reward compatible or neutral outcome option was selected) and a test of difference in responses between OSB groups. A significantly higher percentage of reward-compatible responses compared with reward-incompatible responses was expected, indicating successful instrumental conditioning.

The magnitude of transfer effects in the transfer phase (phase 3) was established as follows. Outcome-specific responses in phase 3 were pooled, calculating a percentage of trials where participants selected the two rewards compatible with their respective Pavlovian cues. Furthermore, the percentage of outcome specific responses were also calculated for selections of the two rewards incompatible with their respective Pavlovian cues. A 2 x 2 mixed ANOVA was used to analyse the difference between outcome-specific responses in low- and high-OSB groups. A higher percentage of compatible transfer-specific effects were anticipated in the high-OSB group.

In phase 3 control conditions, both instrumental response options were incompatible with the Pavlovian cue in reward control and neutral control conditions. Conversely, both instrumental response options were compatible with the Pavlovian cue in the neutral choice condition. Behavioural data relating to the control conditions was not presented in Bray et al. [25] which would allow informed predictions to be made. To speculate, participants might be expected to spread instrumental responses across the two available options in each control condition. A 3 x 2 mixed ANOVA was used to analyse the difference between the proportionally highest options selected across the three control conditions and between low- and high-OSB groups

Between-groups *t-*tests were used to compare low- and high-OSB groups across each of the psychometric measures administered. Pearson’s correlations were used to explore relationships between psychometric measures.

## Results

### Pavlovian-to-instrumental transfer (PIT) task

**Fig 2:**
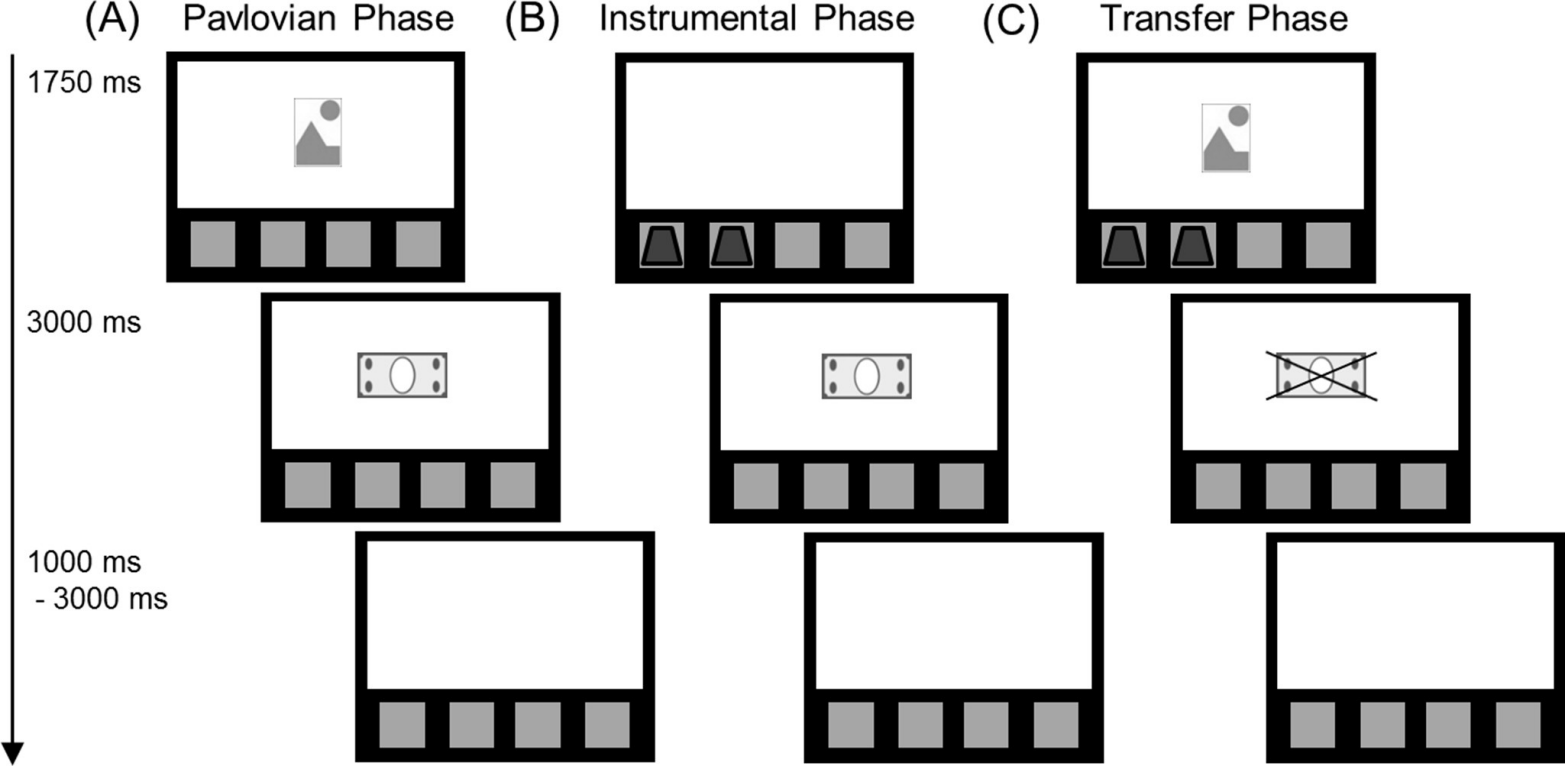
Results of the Pavlovian-to-instrumental transfer task. (A) Phase 1, Pavlovian conditioning. Data shows mean pre- and post-conditioning pleasantness ratings of Pavlovian cues paired with monetary or neutral rewards; (B) Phase 2, instrumental conditioning. Data show mean percentage of trials where the rewarding outcome was selected over the neutral outcome; (C) Phase 3, Transfer phase. Data show mean percentage of responses ± 1 *SE* in each transfer and control condition.

Pavlovian conditioning in phase 1; increases in ratings for cues post-conditioning provide evidence of successful Pavlovian conditioning. A 4 x 2 x 2 ANOVA analysed the pleasantness ratings of Pavlovian cues pre- and post-conditioning between high and low OSB groups. There was a significant main within measures effects for cues (Greenhouse-Geisser corrected; F(2.6,169.8) = 24.31, *p* < .001, η2 = .24) and time (F(1,77) = 24.34, *p* < .001, η2 = .24) and a significant cue x time interaction (Greenhouse-Geisser corrected; F(2.8, 212.7) = 11.13, *p* < .001, η2 = .13). There was no significant between measures main effect of OSB (Greenhouse-Geisser corrected; F(1,77) = 1.2, *p* = .28, η2 = .02) and no significant interactions between OSB and either cues (Greenhouse-Geisser corrected; F(2.6,169.8) = 24.31, *p* = .55, η2 = .01) or time (F(1,77) = .03, *p* = .86, η2 < .01). Post-hoc analyses show increased ratings (Fig 2A) for the cue paired with 100kc (pre: *M* = .23, *SD* = 1.86; post: M = .72, *SD* = 1.65; *t* (78) = -1.874, *p* = .03, η2 = .04, one-tailed) did not reach significance with Bonferroni corrections; increased ratings for cues paired with 500 Kc (pre: *M* = .18, *SD* = 1.81; post: *M* = 1.15, *SD* = 2.17; *t* (78) = -3.77, *p* < .001, η2 < .15) and 1000kc (pre: *M* = .43, *SD* = 2.05; post: *M* = 2.34, *SD* = 2.47; *t* (78) = -5.96, *p* < .001, η2 = .31) monetary values were significant. There was no significant difference between pre- and post-conditioning ratings for the cue paired with the neutral outcome (pre: *M* = -.41, *SD* = 1.68; post: *M* = -.32, *SD* = 1.93; *t* (78) = -.403, *p* = .69, η2 < .01).

Instrumental conditioning in phase 2; the relative response selections provide evidence of response-outcome acquisition. A 2 x 2 ANOVA analysed the proportionate responses related to reward versus neutral outcomes between high and low OSB groups. There was a significant main within measures effect of response selected (F(1,77) = 42.89, *p* < .001, η2 = .36). There was no significant main between measures effect of OSB (F(1,77) = .01, *p* = .94, η2 < .01) and no significant interaction between response selected and OSB (F(1,77) = .03, *p* = .96, η2 < .01). Participants chose (Fig 2B) the response paired with a reward (*M* = .65, *SD* = .19) significantly more often when the alternative associated with a neutral outcome (*M* = .35, *SD* = .19).

Pavlovian to instrumental transfer in phase 3; the specific transfer conditions were aggregated across two trial types and were thus analysed separately to the control conditions. A higher percentage of compatible responses provide evidence of a specific transfer effect. The difference in compatible and incompatible transfer responding between high and low OSB groups was determined using a 2 x 2 ANOVA. There was a significant main within measures effect of transfer type (F(1,77) = 27.85, p < .001, η2 = .27). Both the high-OSB (*M* = .68, *SD* = .26) group and low-OSB group (*M* = .58, *SD* = .26) chose the specific compatible responses (Fig 2C) more often than the specific incompatible responses (low-OSB: *M* = .37, *SD* = .26; high-OSB: *M* = .29, *SD* = .26). However, no significant main between measures effect was found for OSB group (F(1,77) = .48, p = .48, η2 = .01) and there was no significant interaction between transfer type and OSB group (F(1,77) = 2.82, p = .1, η2 = .04).

A 3 x 2 ANOVA analysed the highest proportional response in each of the control conditions between high and low OSB groups. There was no significant main within measures effect of condition (Greenhouse-Geisser corrected; F(1.94,149.24) = 2.85, *p* = .06, η2 = .04), no significant main between measures effect of OSB group (F(1,77) = 1.16, *p* = .29, η2 = .02) and no significant interaction (Greenhouse-Geisser corrected; F(1.94,149.24) = .16, *p* = .85, η2 < .01). The highest proportional response (Fig 2C*)* in the reward control condition was R2 for both low- (*M* = .54, *SD* = .4) and high- OSB (*M* = .55, *SD* = .41) groups, whereas in the neutral control condition, the highest proportional response was R1 for both low- (*M* = .53, *SD* = .31) and high-OSB (*M* = .59, *SD* = .369) groups. Participants in both low- (*M* = .64, *SD* = .28) and high-OSB (*M* = .69, *SD* = .27) groups exhibited a preference for selecting R3 over R4 in the neutral choice control where both responses were associated with the same action-outcome pairing.

### Pair-wise comparisons of psychometric measures

Between-groups *t-*tests were used to compare low- and high-OSB groups across each of the psychometric measures administered (See Table 1.). There was a significant difference between self-report online sexual behaviour between groups (*t* (61.85) = -11.159, p < .001). The high online sexual behaviour group scored significantly higher on measures of sexual compulsivity (*t* (67.33) = -3.497, p = .001) compared to the low online sexual behaviour group. BAS fun (*t* (77) = 2.491, p = .015), BAS reward (*t* (77) = 3.237, p = .002), and BIS (*t* (77) = 2.4, p = .019) were all found to be significantly lower in the high online sexual behaviour group. There was no significant difference in BAS drive between groups (*t* (77) = 1.78, p = .079).

### Correlations

Correlational analysis revealed a significant positive correlation between ISST-OSB and SCS scores (Table 3). There were significant negative correlations between ISST-OSB with BAS drive and with BAS reward responsiveness. Significant negative correlations were also found between SCS and BAS fun-seeking and BAS reward responsiveness. ISST-OSB and SCS each showed a significant negative correlation with BIS scores. There was no evidence of a significant relationship between ISST-OSB and DAST-10 or sMAST scores.

**Table 3:** Pearson’s correlations between psychometric measures.

	SCS	DAST-10	sMAST	BAS D	BAS F	BAS R	BIS
ISST-OSB	.38**	.1	.05	-.24*	-.2	**-.27** ^*^	**-.33** ^**^
SCS		-.02	.12	-.1	**-.31** ^**^	**-.28** ^*^	**-.27** ^*^
DAST-10			.2	-.1	-.02	.12	.12
sMAST				.01	.15	.01	.18
BAS Drive					.15	**.46** ^**^	.09
BAS Fun						**.53** ^**^	**.51** ^**^
BAS Reward							**.56** ^**^

ISST-OSB (Internet Sex Screening Test-Online Sexual Behaviours subscale); SCS (Sexual Compulsivity Scale); DAST-10 (Drug Abuse Screening Test, 10-item form); sMAST (Michigan Alcohol Screening Test, short form); BIS (Behavioural Inhibition System); BAS (Behavioural Activation System). Asterisks denote significance at *p* < .05* or *p* < .01**

## Discussion

The present study was designed to assess whether men who engage in frequent online sexual behaviours differ in their susceptibility to cue-enhanced PIT relative to men who engage in occasional or non-frequent online sexual behaviour. Men in the High-OSB group scored significantly higher than men in the Low-OSB group in the online sexual behaviour subscale of the ISST and in subjective sexual compulsivity. They also scored significantly lower in behaviour inhibition and behaviour activation for fun and reward. Despite these subjective differences, no significant differences were observed between groups in the PIT task, indicating that men in the High-OSB group had no greater susceptibility to cue-induced operant responding than men in the Low-OSB group. Indeed, men in both groups displayed greater pleasantness ratings post-conditioning to cues associated with greater monetary reward relative to neutral cues in Phase 1, and both groups responded significantly above chance for the specific-compatible and neutral cues, and significantly below chance for the specific-incompatible cues. This pattern of results indicates that men in the High-OSB group were not more susceptible to cue-stimulated reward responding than men in the Low-OSB group, providing evidence that frequent online sexual behaviours may not be driven by a general mechanism of positive reward motivation *per se*.

It is generally accepted that addiction is synonymous with a strong reward focus, sensation seeking, and impulsive decision making. For example, BAS dimensions have been more robustly associated with substance abuse and problem drinking [49, 50]. Contrary to prediction, both High-OSB and sexual compulsivity negatively correlated with BAS drive and BAS reward responsiveness in the present study; indicating low motivation in the pursuit of goals with decreased sensitivity to pleasure experienced from rewards. Typically, low BAS drive and BAS reward responsiveness are predictors of depression [40, 63] which is often comorbid with problematic sexual behaviour (see [64]). Indeed, levels of self-esteem have previously been highlighted as risk factors in the development of problematic internet use [65]. Low BAS sensitivity is associated with introversion [40, 66] and frequent pornography use has been reported to increase feelings of loneliness and isolation [67]. Although initial exposure to pornography may be incentivised through positive reinforcement, it has been suggested that problematic online sexual behaviour may develop in some individuals as a means of mitigating negative feelings (see [68, 69]). Research found that high BAS adolescents predicting the development of internet addiction scored lower on BAS dimensions in a follow up study [70]. To speculate, the transition to low motivation state could be influenced by the relative predictability of reward outcome. For example, motivation for rewards can be attenuated in sign-trackers when rewards contingencies become highly predictable [71]. However, individuals persist because internet pornography provides an easily accessible, inexpensive and low effort means of briefly providing negative state relief [72].

It is not known whether men in the High-OSB considered their online sexual behaviour to be problematic. Indeed, viewing pornography online has become part of the normal sexual repertoire [18] and is used predominantly to induce arousal for solitary masturbation and/or sexual encounters with others (see [18]). However, both the use of pornography and sexual compulsivity share comparable negative associations with BIS. High BIS sensitivity provokes feelings of anxiety [73] leading to the abrogation of behaviour to avoid non-rewarding or conflicting outcomes. Low BIS then could indicate disinhibition and low aversion to risky or harmful outcomes [74]. There is some evidence of low BIS in relation to addictive behaviours [50], though findings have proven inconsistent [75, 76]. Taken together, the self-report measure of reward sensitivity suggests that men with High-OSB (and presumably high pornography consumption) may be characterised differently, such that problematic behaviour is more associated with social withdrawal and may provide a means to mitigate negative feelings.

The data from training phases provide evidence of successful conditioning. The pleasantness ratings of cues were generally low. However, ratings of Pavlovian associated cues post-conditioning were proportionally higher with respect to the associated monetary value. The differential ratings were similar to cues associated with soft drinks used in Bray et al. [30] and congruent with research indicating the rewarding nature of monetary values [77]. Furthermore, we showed proportional selection of reward associated Instrumental responses. Similar to Bray et al. [30], participants chose the rewarding option more often when the alternative response was associated with a neutral outcome. Bray et al. [30] found no specific brain regions associated with response in the control condition although behavioural results were not published with which we can draw comparison. Our findings indicate no group differences in responses during the Pavlovian reward control and Pavlovian neutral conditions. However, it is notable that the highest proportional responses in both groups was for the right option associated with the greatest value. In contrast, the highest proportional response in the Pavlovian neutral condition was for the left option associated with the lowest value. Although the Pavlovian associated cues were incompatible, this could be viewed as an attempt to select the best match (neutral closest to 100kc than 500kc; 1000kc closest to 500kc than 100kc). Interestingly, both groups were more likely to select the left response in the neutral choice condition despite both options being equally predicted by the cue-outcome association.

Appetitive Pavlovian cues have previously been shown to influence Instrumental responding [31]. Bray et al. [30] found that cue-outcome pairings modulated responding relative to action-outcome pairings with specific compatible rewards. Although previous research has shown heightened cue reactivity to stimuli associated with compulsive or addictive behaviour (substance, [78]; non-substance, [79]); the present data do not support the idea that high OSB individuals are similarly compulsive. Increased transfer effects related to OSB would have suggested a general mechanism of reward sensitivity beyond sex related cues.

Enhanced PIT effects have previously been shown in relation to individuals with alcohol use disorder [33]. Functional activation of the NAcc predicted the strength of transfer effects and proneness to relapse, highlighting the importance of reward-based learning in addiction [33]. However, PIT has also been demonstrated in a study that failed to show enhanced effects in the high alcohol consumption group [34]. It was noted that participant scores on the alcohol scale were limited in the mid-range and unlikely to have captured individuals exhibiting dependency. Similarly, one possible explanation for lack of transfer effects between OSB groups in our study could be the sample characteristics. While groups used in our study differed in OSB and included individuals in the high OSB group at risk of problematic behaviour, scores on the sexual compulsivity scale were not high; which might explain the apparent lack of difference in transfer effects.

Another study of alcohol dependency found that individuals in the patient group were more likely to suppress a positively reinforced action paired with a negatively valenced cue whilst choosing a negatively reinforced instrumental response when paired with a positively valenced cue [35]. Moreover, PIT effects in this study correlated with measure of trait impulsivity. Failure to suppress a behavioural response owing to the strength of contextual associative cues highlights the dysfunctional choice process through which addicted individuals persist despite negative consequences. While our study found no evidence of transfer effects between OSB groups in relation to appetitive conditioning, further research could investigate negatively valenced associative cues on choice behaviour using PIT.

The relationship between PIT and impulsiveness is posited to be driven by reward sensitivity [35]. High scores on reward sensitivity measures exhibit enhanced appetitive conditioning and a tendency to choose more immediate rewards (impulsive choice) rather than delay gratification for larger later reward (see [73]). BAS drive has also been shown to modulate instrumental responding in relation to higher monetary rewards [80]. Given the negative correlation between BAS drive, BAS reward and OSB, it is perhaps unsurprising that appetitive cues did not translate to enhanced instrumental responding. Altered neural activity in relation to appetitive conditioning has been found in individuals with compulsive sexual behaviour; evidenced by increased amygdala response in addition to decrease in functional coupling between the ventral striatum and pre-frontal cortex [81]. However, such regions implicate impaired control in the development and maintenance of compulsive sexual disorders [81]. Rather than motivated to seek novel and highly stimulating rewards, pornography may simply be a means to satisfy sexual urges and/or attempt to alter negative mood state through previously reinforced behaviour that is not inhibited.

Impaired decision making and sensation seeking are believed to perpetuate addictive behaviours [82, 83] and have been suggested to play a role in compulsive internet pornography use and sexual behaviour [84, 85] although see [86]. Although sensitivity to reward positively correlates with sexually explicit stimuli [51], there is some evidence that problematic users exhibit faster neural habituation to sexual cues [23]. This is congruent with our findings that indicate lower reward sensitivity in the high OSB group. However, an alternative hypothesis is that the high online sexual behaviour group had a mix of individuals with naturally high sexual functioning who may use online pornography for arousal during more frequent episodes of masturbation, relative to the low OSB group, along with other individuals who may do this more compulsively. This raises a challenge for clinicians diagnosing CSBD. It is easy to conceive of high OSB as being indicative of impaired impulse control and decision making, especially when it seems excessive to the individual and/or the individual’s partner(s) based on cultural norms and relationship expectations.

Frequent use of online pornography has been taken de-facto as evidence of so-called “sex addiction” by some clinicians [87]. The evidence presented here indicate that frequent pornography use is not driven by a heightened sensitivity to reward-related cues. Although specific Pavlovian to instrumental transfer was shown, performance was not enhanced in the High-OSB group suggesting that such individuals are not more susceptible to behavioural bias triggered by the motivational influence attributable to reward associative cues. Rather, negative correlations with measures of reward sensitivity and behavioural inhibition imply that frequent pornography use may develop as a disinhibited means to satisfy sexual urges and elicit positive arousal. Kraus et al. [88] have cautioned against over-pathologizing high levels of sexual interest and behaviour in individuals who do not exhibit impaired control over their sexual behaviour and significant distress or impairment in functioning. Clearly, the frequency of viewing pornography online, for example, with or without masturbation, is not itself predicative of problematic sexual functioning. Further research needs to clarify the relationship between reward sensitivity, interpersonal variables (e.g., introversion, sexual self-esteem, perceived impulse control, positive versus negative attitudes about masturbation, etc.), and online sexual behaviour that is truly compulsive and problematic.
